# Cholera Certainties

**Published:** 1882-03

**Authors:** 


### CHOLERA CERTAINTIES.

Eminent medical men have directly opposite opinions on some points connected with cholera. But there is a remarkable unanimity of sentiment among the old school men and the new, allopaths and eclectics, vegetarians and cold water cures, on many facts of a practical character, which it is important for every individual to keep prominently in view until the scourge has passed away.

All agree that cholera prevails most in localities where, in ordinary seasons, the inhabitants suffer from common epidemics, such as diarrhœa, dysentery, fever and ague, and other forms of fever. These manifest themselves in damp places, flat lands, made lands, bottom lands, and at the mouths of rivers. Hence, in cholera times, dry, sandy, high situations are the safest localities, but if persons cannot leave low, damp lands, the next best expedient is to sleep in the highest stories of dwellings, and not descend from them from sundown until after breakfast next morning. If the houses are of but one story, then a blazing fire should be kept for the hour including sunset and sunrise, the family remaining in doors during the interval. The reason of this is, that the cause of these epidemics is an ingredient in the atmosphere which does not necessarily belong to it, hitherto called "miasm," which means an

*emenation*, which seems to arise from the earth, and is more virulent in its ill effects on the surface, and less injurious the higher the ascent from the ground. At about one hundred feet the poison is not supposed to be perceptible. This conclusion was received centuries ago in the East. So also, in 1854, the authorities of London caused observations to be made with this point directly in view; and the truth became patent, that in the exact proportion that houses were elevated above the general level of the city, other things being equal, their inmates were exempt from the scourge, and that at one hundred feet elevation there was scarcely a single case of cholera. The obvious inference then is, that the higher rooms of dwellings should be occupied as chambers.

All admit that filth of neighborhood, of habitation, of chambers, of clothing, of person and of skin, are directly promotive of cholera. The atmosphere is then saturated with impurities. These are taken into the lungs, and thence conveyed into the blood itself, depriving it of its life, making it thick, black and poisonous, dampening the spirits, oppressing the brain, and producing a general feeling of weakness, weariness and fatigue. These impurities are also conveyed into the stomach, mingle with the nutritive materials, and are carried to every portion of the system. Uncleanness also plugs up the pores of the skin, and prevents the escape of that insensible perspiration which is the great scavenger of the body; the ill effects of which may be judged of by the repeated experiments of scientific men, for when an animal is enveloped in an India rubber bag, the nose only protruding, it begins to die in a few hours. The practical lesson taught by these facts is, let the entire body be kept most scrupulously clean. Let the clothing be frequently washed, and aired daily; let every chamber be ventilated and kept dry; damp cellars should be sprinkled with chloride of lime, and all standing water near a building should be conveyed away, and its bed covered with fresh earth or common lime.

Fear will excite a deadly attack of cholera in a few hours. A machinist having seen a comrade die in a blue or collapsed stage, went to work soon after inside a boiler. On emerging into daylight, he noticed that his hands and arms were almost black. He at once took it for granted that this was caused by an attack of the disease, and the shock thus produced ended fatally—he died of cholera symptoms.

It is certain that any violent change in the habits of eating or drinking, while cholera is prevalent, invites the disease, but it is incumbent on all persons to eat regularly of plain, wholesome food, which the stomach can receive and dispose of without inducing indigestion. This should be the universal rule for eating and drinking, as to quantity and quality.

While it is known that cholera is usually ushered in with several thin passages from the bowels, it has been also observed that a failure of the bowels to act for two or three days lays the system, by the necessary reaction, open to a violent and dangerous attack of diarrhœa. Undue action or inaction of the bowels, therefore, is sufficient ground for prompt medical advice.

Getting cool too soon after exercise, which induces visible perspiration on the surface, especially if there is weariness or fatigue, is as certain as anything else to cause a violent attack of cholera when the disease is prevailing. Whenever, then, a person feels uncomfortably warm from exercise, from eating, drinking, or mental excitement, it is the dictate of prudence to retire to a close room, so as not to allow a draught of air to blow on the person. He should not remove any garment for a few minutes, and then should lay them aside one at a time, at intervals. This may seem finical or unduly careful, but it is better to be finical in this matter, than to die in the agonies of cholera a few hours later.

Exposure to the necessity of exercise of any sort in the hot sun from nine A. M. to five P. M. in summer, is likely to invite an attack of cholera to those who are mainly in doors, and it is just as dangerous in a sultry, cloudy or damp day. Persons, then, who are from home, and can be masters of their own movements, at the seaside, springs, hotels or boarding houses, should aim to take their walks, rides, excursions and diversions before nine o'clock in the morning, or else defer them till five in the afternoon, thus remaining cool and quiet during the heat of the day.

To change the dress immediately after coming in, heated and warm, and to throw one's self on the bed, to fall asleep within a minute or two without any covering over the shoulders, or near an open window, as women too often do, has been the means of sending many a sedentary person to the grave in a few hours.

All physicians of all schools agree that the phases of cholera are different in different places, at different seasons, and in different constitutions, requiring a difference of treatment. Dr. Ayre was the most successful physician in Great Britain in the treatment of the disease when it last prevailed in that country. He lost but thirteen per cent. of his patients. He relied mainly on calomel, and objected to all stimulants. Yet, although he gave his formula, it was so unsuccessful in other parts of the country than his own, that it was immediately abandoned. And from almost every country we are now receiving the formulas which have been most successful in each of them, and they very widely differ. This is certainly proof positive that it is safer to rely upon the prescriptions of educated physicians in your own locality; and that taking a prescription on one's own responsibility coming from other localities is very unwise.

But the most indisputable fact of all is this: that as soon as a man notices, in cholera times, that he has a weakening, forcible, thin, painless, light colored discharge from the bowels, he should go directly to bed, send for a physician, remain quiet and warm until the physician arrives, and then submit implicitly to his directions. If a physician cannot be obtained, the man should remain on his bed for two or three days. He can safely eat small quantities of ice to quench his thirst, but should drink nothing but a sip or two occasionally of hot tea. A common woolen flannel, fourteen inches broad, and long enough to double in front, should be bound tightly around the abdomen. He should not eat anything but boiled rice and similar mild food. The probabilities will be ninety-seven per cent. in favor of his recovery.

---

A Pleasant Mouth Disinfectant:—Hypermangate of potassa and hyperoxydate of barium, of each twenty-four grains, to be rubbed up into a mass, with sugar and glycerin, and divided into 144 lozenges. Every ill-smelling mouth will become by their use perfectly odorless.—*Medical Record.*

Gunpowder Marks.—Smear the scorched places with glycerin, by means of a feather, then apply cotton wadding; lastly cover with oil silk. In one case the discoloration was very great, the patient looking more like a mummy than a living being. It entirely subsided in a month by the above treatment.—*London Lancet.*

